# ‘End-stage’ heart failure therapy: potential lessons from congenital heart disease: from pulmonary artery banding and interatrial communication to parallel circulation

**DOI:** 10.1136/heartjnl-2015-309110

**Published:** 2016-12-23

**Authors:** Dietmar Schranz, Hakan Akintuerk, Norbert F Voelkel

**Affiliations:** Pediatric Heart Center, Justus Liebig University Giessen, Virginia Commonwealth University, School of Pharmacy, Richmond, Virginia, USA

## Abstract

The final therapy of ‘end-stage heart failure’ is orthotopic heart, lung or heart-lung transplantation. However, these options are not available for many patients worldwide. Therefore, novel therapeutical strategies are needed. Based on pathophysiological insights regarding (1) the long-term impact of an obstructive pulmonary outflow tract in neonates with congenitally corrected transposition of the great arteries, (2) the importance of a restrictive versus a non-restrictive atrial septum in neonates born with a borderline left ventricle and (3) the significance of both, a patent foramen ovale and/or open ductus arteriosus for survival of newborns with persistent pulmonary hypertension, the current review introduces some therapeutical strategies that may be applicable to selected patients with heart failure. These strategies include (1) reversible pulmonary artery banding in left ventricular-dilated cardiomyopathy with preserved right ventricular function, (2) the creation of restrictive interatrial communication to treat diastolic (systolic) heart failure, (3) atrioseptostomy or reverse Potts shunt in pulmonary arterial hypertension and (4) return to a fetal, parallel circulation by combining atrioseptostomy and reversed Potts shunt with or without placement of a bilateral pulmonary artery banding. While still being experimental, it is hoped that the procedures presented in the current overview will inspire future novel therapeutic strategies that may be applicable to selected patients with heart failure.

## Introduction

End-stage heart failure is the cause of death or need for heart transplantation (HTx) in many cardiovascular conditions.^1^ Heart failure represents a progressive disease with a wide symptom spectrum. Despite existing myocardial damage, initially, endogenous pathophysiological mechanisms will prevent cardiac decompensation and failing of the systemic, morphologically left, right or even single ventricle at rest. Additionally, it is commonly observed that heart failure remains mostly compensated as long as right ventricular (RV) function is preserved. Unfortunately, due to ventriculo-ventricular interaction, systemic ventricular dysfunction will generally have a detrimental impact on the function of the subpulmonary ventricle during long term. Guazzi and Borlaug^2^ emphasise the importance of the lung downstream pressure (left atrial pressure, LAP) as an important contributor to pulmonary artery pressure (PAP). The relationship between mean PAP and LAP is best reflected by the calculated transpulmonary gradient (TPG=mean PAP−LAP) and in particular the diastolic PAP to LAP difference, the so-called diastolic pressure gradient (DPG=diastolic PAP−LAP). Both parameters become even more important in the setting of heart failure: pulmonary hypertension (PH) associated with an increased LAP, but normal TPG (<12 mm Hg) and in particular DPG (<7 mm Hg)—labelled isolated postcapillary pulmonary hypertension—may increase RV afterload and negatively affect right (subpulmonary) ventricular function. During the course of the disease however there may be transition from purely passive pressure elevation to reactive pulmonary vascular disease. The transition from isolated to combined postcapillary pulmonary hypertension is highly variable from patient to patient. Non-reactive or fixed pulmonary arterial hypertension (PAH) is the result of a continuous vascular remodelling, while reactive PH is characterised by a remodelled vasculature that can still dilate.^3^ Chronically increased afterload forces the subpulmonary ventricle to adapt with appropriate hypertrophy but ultimately the RV compensatory mechanisms are exhausted leading to RV failure. As usually RV and left ventricle (LV) are serially connected, advanced right heart failure may lead to a paradoxical underfilling of the LV.^4^ Considering that the right and left hearts do not act in isolation and that LV may be responsible for more than 50% of RV function under healthy conditions,^5^ the changes in right heart pressure and volume affect the left side of the heart and vice versa. This cardiac coupling, referred as ‘diastolic’ ventricular interaction,^6^ is illustrated in the online supplementary video 1a–c.

10.1136/heartjnl-2015-309110.supp1 supplementary video 1a

10.1136/heartjnl-2015-309110.supp2 supplementary video 1b

10.1136/heartjnl-2015-309110.supp3 supplementary video 1c

In summary, left heart-dependent PH might be misinterpreted by focusing exclusively on TPG; the calculated value might be misinterpreted in consideration of the reversibility of PH or in the setting of decision-making for heart or heart-lung transplantation. Therefore, as already emphasised by several other authors,^7^ DPG should be entered into the analysis of PH, because DPG might be low, in spite of a calculated TPG above 15 mm Hg. Generally, the mean and, in particular, the systolic pulmonary pressures are cardiac output (CO)-dependent variables, but the diastolic pulmonary pressure remains quite independent from CO changes.^7^ In addition, considering the margin of error of CO measurements, in particular in congenital heart disease with non-static left-to-right, bidirectional or even right-to-left shunts, we emphasise to use the relationship of the diastolic pressure—pulmonary arterial pressure and systemic arterial pressure (PAPd/SAPd)—ratio as very practical to compare the ‘true’ vascular resistance of both, the pulmonary and systemic circulation. In addition, the precapillary component of the pulmonary vascular circulation can additionally be assumed by analysing DPG.

In LV-dilated cardiomyopathy (DCM), stroke volume remains preserved by LV dilatation on the expense of a high-end systolic volume; the physiopathology of a borderline left ventricle (BLV) and even restrictive cardiomyopathy (RCM) is characterised by CpcPH; the precapillary pulmonary component prevents pulmonary oedema. The properly adapted RV is able over quite some time to maintain filling and pumping. Thus, systemic, or sometimes suprasystemic, pulmonary artery pressures can develop, which correspond to a pulmonary artery banding (PAB) effect that preserves RV systolic function, in particular in the setting of a congenitally restrictive LV-cardiomyopathy (see online supplementary video 1b). However, the disease process is progressive and finally irreversible, resulting in LV, RV or biventricular (BV) failure with subsequent multi-organ failure.

Here we describe established and hypothetical novel percutaneous interventional, surgical open chest and hybrid techniques that may palliate ‘end-stage’ heart failure, with the potential to improve clinical symptoms and for bridging to transplantation; there can be also a chance for recovery, in particular in infants and young children.

**Figure 1:**
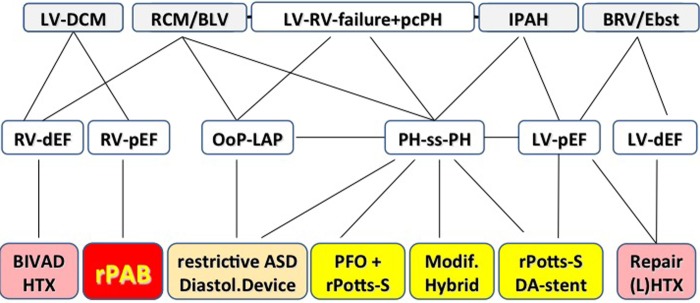
‘End-stage’ heart failure: potential surgical interventional therapies based on left-right heart interactions as described here. BIVAD, biventricular assist device; BLV, borderline left ventricle; BRV, borderline right ventricle; DA, ductus arteriosus; DCM, dilated cardiomyopathy; dEF, depressed ejection fraction; diastol., diastolic; Ebst., Ebstein anomaly; HTx, heart transplantation; IPAH, idiopathic pulmonary hypertension; (L)HTx, (lung) heart transplantation; LV-DCM, left ventricular-dilated cardiomyopathy; LV, left ventricle; LVAD, left ventricular assist device; Modif., modified; OoP-LAP, out-of-proportion left atrial pressure; pcPH, precapillary pulmonary hypertension; pEF, preserved ejection fraction; PFO, persistent foramen ovale; PH-ss-PH, pulmonary hypertension to suprasystemic pulmonary hypertension; rASD, restrictive atrial septum defect; RCM, restrictive cardiomyopathy; rPAB, reversible pulmonary artery banding; rPotts-S, reverse Potts shunt; RV, right ventricle.

Figure 1 summarises our current surgical interventional strategies, which were developed on an individual case-by-case basis, paying attention to natural phenomena like fetal and postnatal transitional circulation and atrioventricular and ventriculo-ventricular interactions.

In our opinion, prior to HTx and Heart-Lung-Transplantation (HLTx) or placement of an LV assist device or biventricular assist device, a repertoire of novel therapeutic surgical interventional options is available that might reduce the need for such procedures.

### Reversible pulmonary artery banding (rPAB)

LV-DCM is characterised by reduced LV ejection fraction (EF), but preserved RV-EF. Idiopathic LV-DCM is the predominant form in infants and young children, while ischaemic DCM predominates in adults. DCM is a myocardial disease with systolic cardiac dysfunction and dilatation.^8^

**Figure 2:**
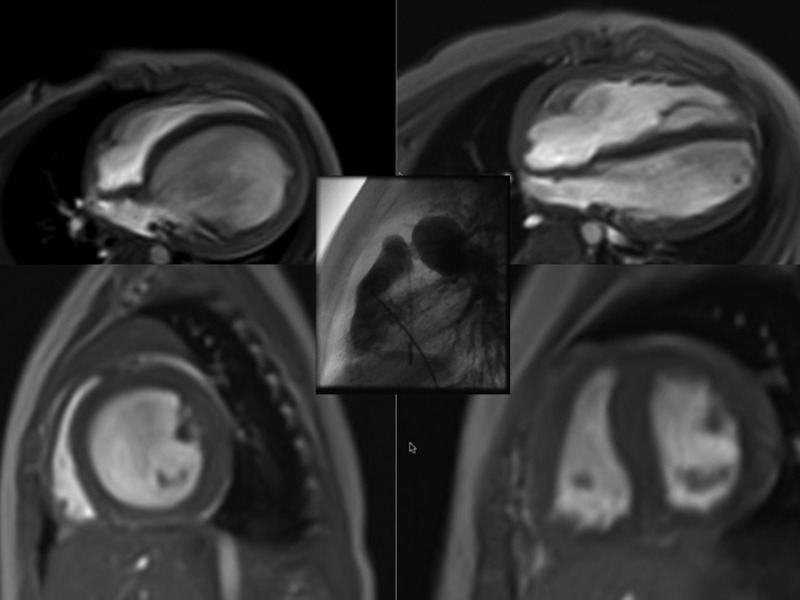
(see, online supplementary video 2a,b) Effects of a reversible pulmonary artery banding (rPAB) on the left ventricle in dilated cardiomyopathy (DCM). Depicted are the MRI of left-sided DCM in four chambers and sagittal views before and in the follow-up after a surgically performed PAB (picture in the middle); the progression of left-ventricular dilatation with its pathological ventriculo-ventricular interaction caused by the morphological change of the left ventricle from ellipsoid to a spherical left ventricle (LV)-form (like pear to apple) lead to a consecutively compressed right ventricular to a banana-shaped chamber associated with an early compromised diastolic inflow. The rationale to place a surgically rPAB is to induce a leftward mechanical shift of the interventricular septum back to the LV with a normally shaped left ventricle potentially resulting in an improved LV function.

Considering patients with a systemic RV, the long-term outcome is directly related to a balanced ventricular interaction and the competence of the systemic atrioventricular valve.^9^ Dilatation of the systemic ventricle is prevented by subpulmonary (left) ventricular outflow tract obstructions or even significant PH. Surgical rPAB may be used, if such an obstruction is absent.^10^ In congenitally corrected transposition of the great arteries (CCTGA), rPAB has been used for retraining of subpulmonary LV-DCM,^10^ and as a preventative strategy in newborns.^11^ Considering favourable results of PAB in children with CCTGA, in Giessen^12^ we also applied surgical rPAB to treat LV-DCM with preserved RV-EF (figure 2 and online supplementary video 2a,b). The entry criteria for using rPAB in the setting of LV-DCM have been recently published.^12^ The proposed mechanisms of rPAB action are summarised in box 1: it is postulated that the efficacy of rPAB correlates negatively with the patient's age. The potential for myocardiocyte recovery and myocardiocyte repopulation may be greatest in infancy.^13^ Hypothetically, even in adults an adjustable PAB might be able to induce in certain patients a less rapid, but nevertheless significant improvement of LV systolic impairment, but this remains unproven at present. Sufficient RV adaption to PAB seems to be age-dependent not at least by the chance of young children to grow in PAB, which may prevent the need for rebanding.^12^ Animal models demonstrated that chronic PAB increases the myocytal size during RV-hypertrophy and induces a cell growth-directed gene expression pattern, accompanied by a maintained capillary network.^14^ Additionally, the adaptive response of the cardiac pressure overload is associated with an IGF release and fibroblast expression of the transcription factor KLF-5.^15^ In infants and young children with LV-DCM, rPAB improved LV-EF and decreased highly significantly left ventricular end-diastolic diameter (LVEDD) and brain natriuretic peptide (BNP) blood levels.^12^ The surgical technique of PAB also allows percutaneous catheter-based gradual debanding.^12^

Box 1 **Mechanisms of reversible PAB in LV-DCM**

1. Increase of RV contractility (Anrep effect), wall stress and isovolemic contraction

2. Stimulation of RV hypertrophy and matrix remodelling

3. Improvement of RV diastolic inflow characteristics

4. Leftward shift of the IVS and restoration of electromechanical RV-LV synchrony

5. Reduction of LVED volume with reshaping from spherical to ellipsoid morphology

6. Reduction of LV-filling dynamics (preload) and subsequent LVED pressure

7. Improvement of LA–LV interaction by reduction of left atrial volume overload

8. Restoration and improvement of MV regurgitation and LV ejection fraction

9. LV reverse remodelling (co-hypertrophy) by favouring endogenous repair potentials

IVS, interventricular septum; LA, left atrium; LV, left ventricle; LV-DCM, left ventricular-dilated cardiomyopathy; MV, mitral valve; PAB, pulmonary artery banding; RV, right ventricular.

10.1136/heartjnl-2015-309110.supp4 supplementary video 2a

10.1136/heartjnl-2015-309110.supp5 supplementary video 2a

The current results, partially published,^16^ can be seen as ‘proof-of-principle’ data obtained in currently 35 infants and young children: 31 procedures were successful, while 4 children did not show any response. Thus, rPAB in LV failure may provide a novel alternative for ‘bridge-to-transplant’ or destination therapy in children with advanced LV-DCM; in addition, nowadays there are also some successful case experiences with PAB in LV-DCM in several other centres worldwide. Considering the fact, that randomised studies in Europe and the USA are planned for rPAB in infants with DCM, we want to point out that the surgical rPAB needs to be paired with an improved anticongestive therapy.^17^ The endogenous neurohumoral activation, which occurs in all forms of heart failure, should be inhibited, especially in severe forms of LV-DCM; the myocardiocytes and cardiac interstitium need to be protected against the additional mechanical stress of PAB. Taking the neurohumoral activation in patients with severe DCM and considering the differences of the β-adrenergic receptor pathophysiology between paediatric and adult patients with DCM, in our institution^17^ all infants and children undergoing rPAB are being treated with a β1-specific β-blocker (bisoprolol), a tissue ACE inhibitor (lisinopril) and a mineralocorticoid blocker (spironolactone), while we avoid other diuretics to the extent that is possible. Clearly, long-term risks and benefits need to be analysed in a multicentre study. Additionally, it should prove if this approach can be expanded to older children suffering LV-DCM. In summary, it is hypothesised that rPAB leads to LV recovery and diminishes the need for mechanical circulatory assist devices or HTx in selected patients and should be considered.

### Creation of an interatrial communication for diastolic (systolic) heart failure

**Figure 3:**
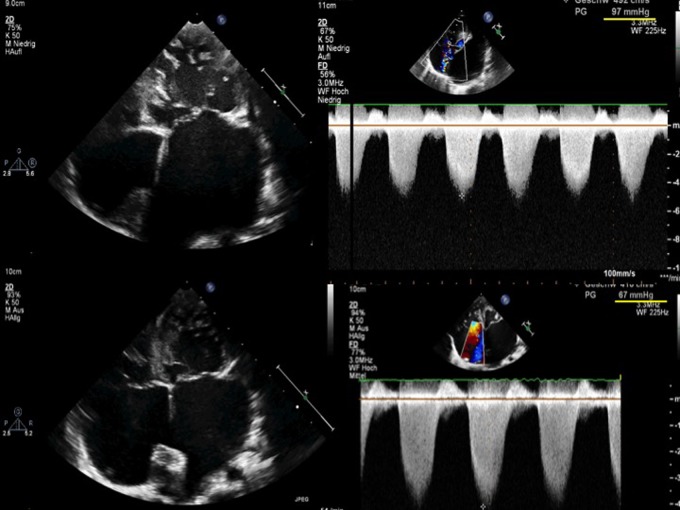
Restrictive cardiomyopathy (RCM), effects of a restrictive interatrial communication. Two-dimensional (2-D) echocardiography (apical four-chamber view) and continuous wave (CW)-Doppler measurements (tricuspid regurgitation, TR) of a toddler with RCM referred for heart transplantation (HTx); the 2-D echocardiography shows massive left atrial enlargement associated with a ‘out-of-proportional’ left atrial pressure related pulmonary hypertension at a suprasystemic level (systolic pressure gradient of 97 mm Hg, upper panel). The creation of a restrictive atrial septum defect, by placing a stent within the perforated atrial septum, led to a decreased left atrial dimension/pressure as well as reduced pulmonary artery pressures (lower panel). Placement of an assist device could be avoided, a successful HTx performed.

Heart failure with preserved ejection fraction is attributed to diastolic dysfunction, which ranges from discrete failure of myocardial relaxation to significant anatomical limitations of the size of the systemic ventricle.^18^ The prominent characteristic of diastolic incompetence of the systemic ventricle is a dilated left atrium independent of a pre-existing atrioventricular concordance or discordance. Advanced ageing hearts acquire commonly a diastolic dysfunction with preserved LV-EF.^19^ LV RCM leads to an impaired ventricular filling, which underlines the importance of the systolic to diastolic duration ratio.^20^ Patients with LV RCM, but morphologically not affected RV, remain clinically stable at rest despite excessive PH values.^21^ Thus risk stratification and the decision to list a patient for HTx become problematic and need to be assessed on an individual basis.^22^ Medical therapy options are limited (mostly to diuretics) and do not improve outcome. Of the presently used treatment options so far, only the mineralocorticoid receptor blocker, spironolactone, showed a moderate, but significant improvement of heart failure with preserved EF.^23^ On the contrary, several transcatheter or surgical interventional hybrid strategies might be an additional option in severe diseased diastolic dysfunctional left hearts, independent if these are caused by LV-RCM, BLV or congenital left-heart defects like a Shone complex. Based on our experiences with neonates born with a hypoplastic left-heart complex,^24^ the use of fenestration in the Fontan circulation and the effects of a restrictive left-to-right shunt in patients with RCM and DCM, we hypothesise that a defined interatrial communication might improve congestive symptoms and reduce the incidence of atrial tachyarrhythmia (eg, atrial fibrillation) in these settings. Such a restricted atrial septum defect with an age-dependent width between 4 and 10 mm can be created by a transcatheter atrioseptostomy followed by passive gradual ballooning or by placement of a slightly diabolo-shaped stent (figure 3). In future, several ‘diastolic devices’ with a predefined hole will become available. These should prevent re-occlusion and allow left-heart interventions, if necessary. These measures of a restrictive, left-to-right shunt are able to reduce the degree of left atrial hypertension. Left atrial decompression improves the atrioventricular interaction and global haemodynamic function; by reducing left atrium pressure and enlargement may be the incidence of atrial fibrillation is influenced, too. In addition, secondary PH should be reduced by direct impact on the pulmonary congestion.

Considering our currently only partially published institutional experience of percutaneous left atrial decompression,^25^ creating a restrictive atrial septum defect (rASD) is safe and technically feasible at any age. Restriction of the interatrial communication preserves an adequate filling pressure for diastolic and/or systolic dysfunctional systemic ventricles, but clinical symptoms related to the ‘out-of-proportion’ left atrial and pulmonary pressures can significantly be influenced.

### Back to a ‘fetal’ parallel circulation: atrioseptostomy, reversed Potts shunt or both

A reverse Potts shunt has been described as an alternative for lung transplantation in selected children with suprasystemic idiopathic PAH.^26^ The anastomosis between the left pulmonary artery and descending aorta allows a right-to-left shunt and thereby decompression of a failing RV combined with an improvement of the systemic circulation on expense of oxygen desaturation of the lower body part if pulmonary arterial pressures are suprasystemic. However, in contrast to a right-left shunting intracardiac defect, oxygen desaturation of the upper part of the body is avoided. Recently, transcatheter techniques have been reported for re-establishing a right-to-left shunting duct in patients with suprasystemic PH of various etiologies,^27^ and even for the creation a de novo communication between the left pulmonary artery and descending aorta.^28^

**Figure 4:**
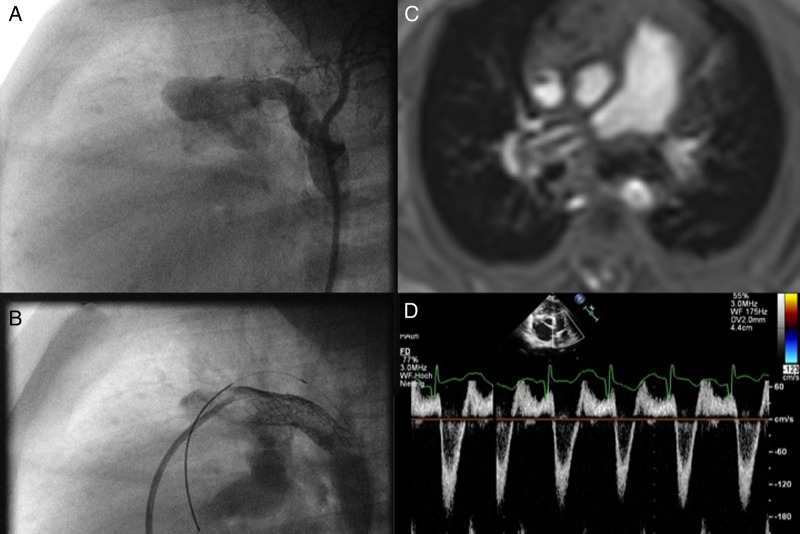
(A–D) The need for a valved Potts shunt. (A) shows a surgically performed reversed ‘Potts’ shunt with a dimension of 6 mm; in the follow-up, the polytetrafluorethylen (PTFE)-shunt was dilated by placing a 8 mm Formula Stent (B); but despite a bilateral pulmonary banding, which is depicted on MRI (C), the Doppler echocardiography (D) shows a systolic right-to-left, but a diastolic left-to-right shunt flow.

Based on the experience with ‘Hybrid approach’ in neonates with a morphologically hypoplastic left heart,^29^ we believe that a comparable strategy, back to a parallel ‘fetal-like’ circulation, might offer an option for selected patients with ‘end-stage’ left-heart failure associated with CpcPH.^30^ ^31^ The creation of a rASD decompresses an ‘out-of-proportion’ LAP and leads—depending on the atrial pressure differences—to a left-to-right shunt. In combination with a right-to-left shunting pulmonary-to-systemic communication, BV function may improve by unloading and economising both sides of the heart. As suprasystemic pulmonary and RV pressure decreases to a systemic level, the heart becomes unloaded by a decreased LV preload and a resynchronised ventricular interaction. This may be accompanied by improved systemic oxygen delivery potentially leading to improved New York Heart Association functional class. Such a duct-like Eisenmenger's physiopathology does not preserve highly oxygenated coronary and cerebral blood flow but the combination with a left-to-right shunting rASD could avoid extreme oxygen desaturation of the lower body part, too. The availability of a valved Potts shunt conduit would allow an earlier intervention via transcatheter or surgical techniques in patients with an idiopathic or left heart-dependent PAH. The intervention could be performed even in terms of a diastolic PAP:SAP ratio of <1. Furthermore, in case of improvement of pulmonary pressures after Potts shunt creation, a diastolic left-right shunt would be avoided. In particular in selected young patients, achievement of a parallel circulation requires the combination of an interatrial, left-right shunt with a reverse Potts shunt, and the addition of bilateral PAB^31^; however, in spite of such bandings, a diastolic left-to-right shunt might occur in particular when the haemodynamics improve (figure 4). This highlights some of the potential dangers associated with re-ducting or creating a reversed Potts shunt, emphasising the need for careful assessment in particular if cardiac failure is associated with CpcPH.

Although speculative at present, ‘Hybrid strategies’ might have a survival benefit even in adolescents with a severe diastolic left-heart failure. Timing of such a comprehensive approach in the setting of a dysfunctional global circulation might, however, be difficult. We argue that a disproportionate postcapillary PH and ensuing RV failure necessitating listing for heart-lung transplantation might be one criterion, favouring a hybrid approach that includes percutaneous atrioseptostomy with or without device implantation and a surgically created or transcatheter-created reversed Potts shunt, in some together with bilateral pulmonary banding, although this approach has to be still regarded as experimental at present.

In conclusion, this article provides insight into some novel methods for managing selected patients with heart failure. These procedures have still to be regarded as experimental and should only be regarded as a potential adjunct to management in accordance to the current heart failure guidelines.^32^ The procedures are inspired by our work with children with congenital heart disease and the pathophysiological insights gained in this setting. It is hoped that the provided figures and online supplementary video represent informative examples for the concepts discussed. Some congenital conditions may serve as a model disease that may eventually lead to novel therapy options for heart failure. Especially in adults with acquired heart failure, these treatments currently remain largely untested and require further studies.
